# Silencing LINC00987 ameliorates adriamycin resistance of acute myeloid leukemia via miR-4458/HMGA2 axis

**DOI:** 10.1186/s13062-024-00490-1

**Published:** 2024-06-24

**Authors:** Yue Liu, Xiao-ya Zhu, Li-li Liao, Zhan-hui Zhang, Tao-sheng Huang, Ling Zhang, Xi-wen Jiang, Yi Ma

**Affiliations:** 1 Institute of Biomedicine, Department of Cellular Biology, Jinan University, No. 601 Huangpu Ave West, Shipai Street, Tianhe District, Guangzhou, Guangdong 510632 China; 2 State Key Laboratory of Virology, College of Life Sciences, Wuhan University, Wuhan, 430072 China; 3 Research Center of Medical and Pharmaceutical Bioengineering, Minstry of Health, Guangdong Province Nucleic Acid Molecular Diagnostics Engineering Technology Research Center, Daan Gene Co Ltd, Guangzhou, 510663 China; 4 Department of Hematology, The Third Affiliated Hospital of Sun Yat-sen University, No. 600 Tianhe Road, Shipai Street, Tianhe District, Guangzhou, 510630 China; 5 National engineering research center of genetic Medicine, Key laboratory of Bioengineering Medicine of Guangdong Province, Guangzhou, 510632 China; 6 The National Demonstration Center for Experimental Education of Life Science and Technology, Jinan University, Guangzhou, 510632 China

**Keywords:** Acute myeloid leukemia, Adriamycin, Long non-coding RNA, microRNA

## Abstract

**Background:**

Most patients with acute myeloid leukemia (AML) eventually develop drug resistance, leading to a poor prognosis. Dysregulated long gene non coding RNAs (lincRNAs) have been implicated in chemoresistance in AML. Unfortunately, the effects of lincRNAs which participate in regulating the Adriamycin (ADR) resistance in AML cells remain unclear. Thus, the purpose of this study is to determine LINC00987 function in ADR-resistant AML.

**Methods:**

In this study, ADR-resistant cells were constructed. LINC00987, miRNAs, and HMGA2 mRNA expression were measured by qRT-PCR. P-GP, BCRP, and HMGA2 protein were measured by Western blot. The proliferation was analyzed by MTS and calculated IC50. Soft agar colony formation assay and TUNEL staining were used to analyze cell colony formation and apoptosis. Xenograft tumor experiment was used to analyze the xenograft tumor growth of ADR-resistant AML.

**Results:**

We found that higher expression of LINC00987 was observed in AML patients and associated with poor overall survival in AML patients. LINC00987 expression was increased in ADR-resistant AML cells, including ADR/MOLM13 and ADR/HL-60 cells. LINC00987 downregulation reduces ADR resistance in ADR/MOLM13 and ADR/HL-60 cells in vitro and in vivo, while LINC00987 overexpression enhanced ADR resistance in MOLM13 and HL-60 cells. Additionally, LINC00987 functions as a competing endogenous RNA for miR-4458 to affect ADR resistance in ADR/MOLM13 and ADR/HL-60 cells. HMGA2 is a target of miR-4458. LINC00987 knockdown and miR-4458 overexpression reduced HMGA2 expression. HMGA2 overexpression enhanced ADR resistance, which reversed the function of LINC00987 silencing in suppressing ADR resistance of ADR/MOLM13 and ADR/HL-60 cells.

**Conclusions:**

Downregulation of LINC00987 weakens ADR resistance by releasing miR-4458 to deplete HMGA2 in ADR/MOLM13 and ADR/HL-60. Therefore, LINC00987 may act as the therapeutic target for treating chemoresistant AML.

**Supplementary Information:**

The online version contains supplementary material available at 10.1186/s13062-024-00490-1.

## Background

Acute myeloid leukemia (AML), the most common subtype of leukemia, is the highly heterogeneous hematopoietic malignancy with a high proportion of AML in children and the elderly [1]. The main treatments for AML are chemotherapy and stem cell transplantation, which have greatly improved over the last decades [2, 3]. Many chemotherapeutic drugs, including Adriamycin (ADR), are currently used to treat AML [4, 5]. Unfortunately, approximately 25% of patients still suffer from multidrug resistance, leading to disease recurrence, poor chemotherapy efficacy, and a lower survival rate in AML patients [6]. Multidrug resistance was associated with the overexpression of breast cancer resistance protein (BCRP) and P-glycoprotein (P-GP) protein in cancer [7]. Compared to the untreated patients with AML, BCRP and P-GP protein were increased in relapsed patients with AML [8]. High BCRP and P-GP protein had been closely related to poor prognosis because of chemoresistance [9]. Thus, multidrug resistance is still a major obstacle for treating AML. Therefore, it is necessary to discover the molecular mechanisms of chemoresistance in AML and identify a revolutionary target to reverse chemoresistance in AML.

Dysregulation of lncRNAs (such as lncRNA CRNDE, KCNQ1OT1, and ZFAS1) is implicated in chemoresistance in AML [10–13]. Long intergenic non coding RNAs (lincRNAs), a type of lncRNA, are emerging as key regulators in cancer development. LincRNA can serve as a prognostic marker for AML [14]. LincRNAs can enhance the expression and biological functions of miRNA targets by acting as competitive endogenous RNAs (ceRNAs) to miRNA sponges. LINC00675 promotes the AML malignancy via sponging miR-6809 to positively regulate CDK6 expression [15]. LINC00599 attenuates AML malignancy by sponging miR-135a-5p [16]. More importantly, lincRNA is closely related to tumor resistance. LINC02321 promotes cisplatin resistance in bladder cancer [17]. LINC-PINT reduced the cisplatin chemoresistance of laryngeal carcinoma cell via sponging miR-425-5p to positively regulate PTCH1 and SHH protein [18]. In addition, LINC00239 overexpression promoted chemoresistance against doxorubicin in AML cells [19]. Unfortunately, the role and mechanism of lincRNAs in ADR-resistant AML is still not complete understood.

In the present study, lincRNAs associated with poor prognosis of leukemia were analyzed using the GEPIA website. Then, ADM-resistant acute leukemia cells were established, and lincRNA expression in AML and corresponding ADM-resistant AML cell lines was measured. LINC00987 expression was higher in ADM-resistant AML cells, then the effect of LINC00987 on ADM-resistant in AML cell lines was studied. Then, the miRNAs sponged by LINC00987 and their target genes were analyzed. Furthermore, the mechanism underlying LINC00987/miRNA/target gene-regulated ADR resistance was explored. This study aimed to identify a novel treatment target for attenuating ADR resistance in AML cell lines.

## Materials and methods

### ADR-resistant AML cell

MOLM13 and HL-60 were purchased from Zqxzbio (Shanghai, China) and cultured in complete media ZQ-905 and ZQ-218 (Zqxzbio). ADR-resistant cells were induced by a low-concentration gradient increase. Briefly, the sensitivity of MOLM13 and HL-60 to ADR was detected. MOLM13 first incubated with 0.01 µmol/L ADR for two week, then Incubated with 0.02, 0.04, 0.08 µmol/L ADR for one week. When using 0.16 µmol/L incubation, MOLM13 cells showed significant death. Hence, 0.08, 0.12, and 0.16 µmol/L ADR were further used to treat for 2 weeks. When using 0.20 µmol/L ADR incubation, a large number of MOLM13 cells were dead. And the experiment did not progress after continuous cultivation for 3 times. Hence, 0.16 µmol/L ADR was used to maintain cultivation for 4 weeks for subsequent experiments. Similarly, when using 0.28 µmol/L ADR incubation, a large number of HL-60 cells were dead. And the experiment did not progress after continuous cultivation for 3 times. Hence, 0.24 µmol/L ADR was used to maintain cultivation for 4 weeks for subsequent experiments. ADR-resistant cells were constructed, and the IC50 was calculated.

### Proliferation

For proliferation measurements, 1 × 10^4^ transfected cells were seeded in 96-well plates for culturing. Then 10 µl AQueous One Solution reagent (Promega) was added at 0, 24, 48, and 72 h. The optical density at 490 nm (OD490 nm) was measured after cultivation for 4 h. Cell viability was calculated using the following formula: (OD 490 nm in experiment group/OD 490 nm in control group × 100%). The OD_490_ was measured after different concentrations of ADR treatment at 0, 24, 48, and 72 h to calculate IC50. The assays were performed in duplicate.

### Transfection

LINC00987 (NR_036466) and high mobility group AT-hook 2 (HMGA2, NM_003483) cloned into the pcDNA3.1 vector to establish the LINC00987 and HMGA2 overexpression vectors (pcDNA-LINC00987 and pcDNA-HMGA2, GENERAL bio, Guangzhou, China). Empty pcDNA3.1 vector (pcDNA-Empty) was used as a negative control. Small interfering RNAs (siRNAs) against LINC00987 (si-LINC00987), and a scramble negative control (si-NC), were chemically synthesized by Ribobio (Guangzhou, China). The miR-4458 inhibitor and negative inhibitor control (miR-NC inhibitor) were purchased from Ribobio. All transfections were performed by electroporation using an Entranster™-E (Engreen, Beijing, China). To elucidate the impact of LINC00987 on ADM-resistance, pcDNA-LINC00987 and pcDNA-Empty were transfected into MOLM13 and HL-60 cells, and si-LINC00987 and si-NC were transfected into ADR/MOLM13 and ADR/HL-60. To elucidate the relationship between LINC00987, miR-4458, and HMGA2, si-LINC00987 and miR-4458 inhibitor (or miR-NC inhibitor) or si-LINC00987 and pcDNA-HMGA2 (or pcDNA-Empty) were co-transfected into the ADR/MOLM13 and ADR/HL-60.

### RT-qPCR

For RNA extraction, ADR/MOLM13, ADR/HL-60, MOLM13, and HL-60 cells were collected and isolate total RNA using TRIzol reagent (Invitrogen). Reverse transcription was carried out using the EasyScript First-Strand cDNA Synthesis SuperMix kit (TransGen Biotech, Beijing, China). The qPCR was prepared using SYBR Green qPCR SuperMix (YEASEN, Shanghai, China), according to manufacturer instructions, which was performed using the ABI PRISM® 7500 Sequence Detection System (Applied Biosystems, Foster City, CA, USA). β-actin and U6 were analyzed as internal control genes for lincRNAs and miRNAs, respectively. The primers (5′–3′) are shown in Supplementary Table 1. The assays were performed in triplicate.

### Soft agar colony formation assay

First, 1.2% and 0.7% agarose solutions were prepared. Then, 1.2% agarose solution and 2 × complete medium were mixed and added to 96-well culture plates as the base agar layer. Next, 0.7% agarose solutions and 2 × complete medium were mixed, and ADR/MOLM13, ADR/HL-60 cells, MOLM13, and HL-60 cells were suspended in complete medium and mixed with 0.7% agar. Then, 1 × 10^4^ cells/well were seeded into 96-well culture plates and solidified on the base agar layer. The 96-well culture plates were incubated at 37 °C in a 5% CO_2_ atmosphere for 7 days. Complete medium (200 µL was added every two days during the incubation period. After culturing for 7 days, the number of cell clones was counted by imaging at 100x magnification using a LEICA microscope. The assays were performed in triplicate.

### TUNEL staining

TUNEL staining was used to detect cell apoptosis. Briefly, the cell slice was incubated at 37 °C for 30 min and washed by PBS. The DeadEnd™ Fluorometric TUNEL System (G3250, Promega) was added to the slice, which was incubated to 1 h in 37 °C and dark condition. Then the slice was washed with PBS and dried for mounting. TUNEL staining was observed using a digital pathological section (fluorescence) scanning analyzer. The assays were performed in triplicate.

### Western blot

Total protein (30 µg per lane) was isolated using RIPA buffer. After 12% and 8% SDS-PAGE, the proteins were transferred onto PVDF membranes. Membranes were blocked, incubated with primary antibodies, The primary antibodies used were as follows: rabbit monoclonal anti-P glycoprotein (P-GP; 1:1000, ab170904, Abcam, San Diego, CA, USA), breast cancer resistance protein (BCRP, 1:1000, ab207732, Abcam), HMGA2 (1:1000, ab246513, Abcam), and anti-GAPDH (1:10000, AF2071, Affinity, San Diego, CA, USA). Horseradish peroxidase-conjugated secondary goat anti-mouse IgG (G-21,040, Ebioscience, 1:1000) was incubated at 1 h in 25 °C. Protein abundance was shown by enhanced chemiluminescent reagent (Thermo Scientific Pierce, Rockford, IL, USA).

### Xenograft tumor

A total of 20 Female 5-week-old BALB/c nude mice (13–15 g) were from the Guangdong Medical Laboratory Animal Center (SCXK(Yue)2018-0002, Guangzhou, China) and were fed with autoclaved standard laboratory feed and given free access to sterile drinking water under SPF conditions (25 ℃ and 12-h light/dark cycle). Lentiviruses carrying sh1-LINC00987 and empty lentiviruses (Laideliankang, Guangzhou, China) were infected into the ADR/MOLM13 and ADR/HL-60 cells. To screen cells infected with lentivirus, ADR/MOLM13 and ADR/HL-60 cells were cultured in a complete culture medium containing 4 µg/ml puromycin at 48 h after infection, and then the medium was changed every 2 days. Simultaneously, ADR/MOLM13 and ADR/HL-60 cells which had not infected with lentivirus were used as control experiments. After all ADR/MOLM13 and ADR/HL-60 cells in the control experiments were dead, ADR/MOLM13 and ADR/HL-60 cells which infected with lentivirus were cultured in a complete culture medium with 1 µg/ml puromycin. The stable cell lines were obtained after continued to pass on for 3 generations. Then the stable cells were collected and made into single cell suspension at a density of 5 × 10^6^ cells/mL, 100 µL of which was inoculated subcutaneously into the dorsal root of the right hind limb of nude mice. Nude mice were randomization divided into four groups (*n* = 5): ADR/MOLM13-sh-NC, ADR/MOLM13 + sh1-LINC00987, ADR/HL-60-sh-NC, and ADR/HL-60 + sh1-LINC00987. ADR solution (10 mg/kg) was intravenously administered every 5 days. The mice were euthanized at 30 day after inoculation, and the subcutaneous tumor was removed. The length and width of the subcutaneous tumor was measured by the digital caliper (Guanglu, Guilin, China), and the volume of subcutaneous tumor was calculated: volume = (length × width × width)/2. The animal experiments were approved by the GIBH Application to Use Animals for Research (A5748-01) in accordance with the Basel Declaration.

### Luciferase reporter and Ago2-RIP assays

The psi-CHECK2 luciferase reporter gene vectors which cloned the wild-type LINC00987 (WT LINC00987), wild-type HMGA2 mRNA 3′-UTR (WT HMGA2), mutant LINC00987 (MUT LINC00987; All sites that bind to miR-4458 have undergone mutations), mutant HMGA2 mRNA 3′-UTR (MUT HMGA2; All sites that bind to miR-4458 have undergone mutations) (GENERAL bio, Guangzhou, China) were transfected with into ADR/MOLM13 and ADR/HL-60 cells. And miR-4458 mimic or NC mimic was co-transfected with into ADR/MOLM13 and ADR/HL-60 cells. After 48 h, Renilla (R) and firefly (F) luciferase activity were was monitored using the Dual-Luciferase Assay kit (Promega) and calculated R/F. AGO2-RIP assay was performed using EZ-Magna RIP RNA-Binding Protein Immunoprecipitation Kit (Merck Millipore, Bedford, MA, USA) following the manufacturer’s instructions. Magnetic beads were conjugated with human anti-Ago2 antibody or anti-IgG antibody (negative control). In addition, LINC00987and miR-4458 expression was measured by qRT-PCR.

### Bioinformatics analysis

To analyze the role of lincRNA in prognosis judgment and drug resistance, the top 100 differentially survival genes were analyzed in “Most Differential Survival Genes” module using the ‘Survival Analysis’ of GEPIA (http://gepia2.cancer-pku.cn/#general). In addition, the relationship between LINC00987, LINC00152, and LINC00982 expression with LAML survival were analyzed in “Survival Plots” module using the ‘Survival Analysis’ of using GEPIA. And LINC00987, LINC00152, and LINC00982 expression in LAML patients were analyzed in “Box Plots” module using the ‘Expression DIY’ of GEPIA. The top 100 genes that had a similar expression trend to LINC00987 in AML were analyzed using the ‘Similar Genes Detection’ of using GEPIA. Next, miRcord (http://www.mircode.org/index.php) and LncBase Predicted v2 (https://dianalab.e-ce.uth.gr/html/diana/web/index.php?r=lncbasev2%2Findex-predicted) were used to analyze the potential miRNA sponges of LINC00987. Finally, Starbase 3.0 (https://rnasysu.com/encori/index.php), miRBD (https://mirdb.org/), and Targetscan 8.0 (https://www.targetscan.org/vert_80/) were used to analyze the potential targets of miR-4458. The clinical correlation of LINC00987 vs. miR-4458 or HMGA2 v miR-4458 in AML patients were analyzed by Starbase 3.0 (https://rnasysu.com/encori/index.php).

### Statistical analysis

All data in the bar graph are presented as mean ± standard deviation. One-way analysis of variance (ANOVA) followed by Turney’s test was used to analyze statistical differences between more than 2 groups using SPSS software (version 19.0; SPSS Inc., Chicago, IL, USA).

## Results

### LINC00987 expression was upregulated in ADR-resistant AML cells

**Fig. 1:**
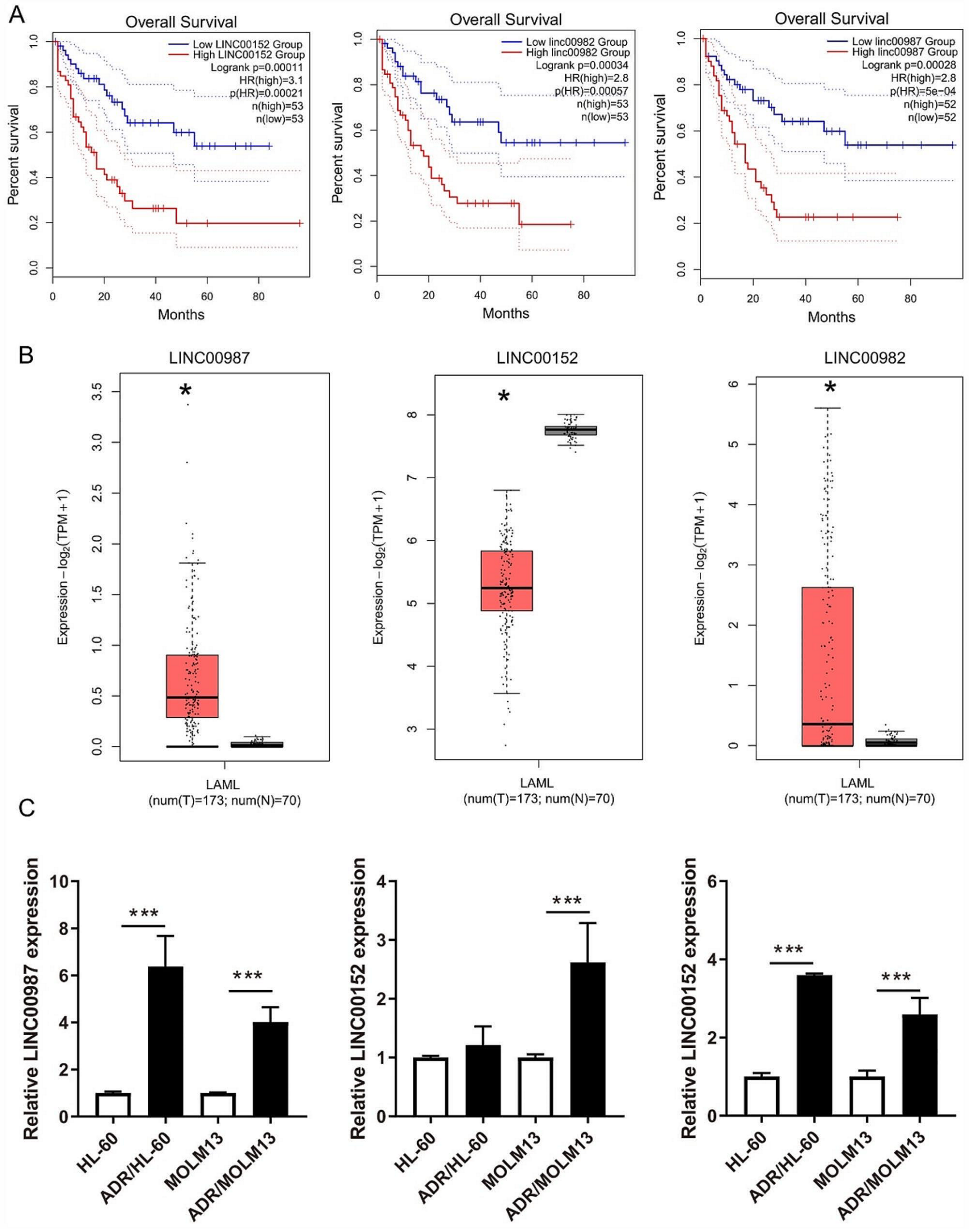
LINC00987 expression was higher in AML patients and associated with poor overall survival. (**A**) The relationship between LINC00987, LINC00152, and LINC00982 expression with LAML survival were analyzed using GEPIA. (**B**) LINC00987, LINC00152, and LINC00982 expression in LAML patients were analyzed via GEPIA. (**C**) LINC00987, LINC00152, and LINC00982 expression in MOLM13, HL-60, ADR/MOLM13, and ADR/HL-60 cells were measured via RT-qPCR. ***P < 0.001

To analyze the role of lincRNA in prognosis judgment and drug resistance, the top 100 differentially survival genes were analyzed using GEPIA. Then it found that three lincRNAs (LINC00987, LINC00152, and LINC00982) were associated with poor overall survival in AML patients (Fig. 1A). Higher expression of LINC00987 and LINC00982 was observed in AML patients than in normal individuals, while lower LINC00152 expression was observed (Fig. 1B). To detect LINC00987, LINC00152, and LINC00982 expression in ADR-resistant AML cells, ADR/MOLM13 and ADR/HL-60 cells were constructed. More interestingly, compared to the corresponding AML cells (MOLM13 and HL-60), the IC50 of the drug-resistant AML cell lines (ADR/MOLM13 and ADR/HL-60) was significantly increased (Supplementary Fig. 1). The results showed that the ADR-resistant AML cells were successfully constructed. Additionally, LINC00987 and LINC00982 expression was significantly increased in ADR/MOLM13 and ADR/HL-60 cells compared with that in MOLM13 and HL-60 cells, especially the expression of LINC00987 (Fig. 1C). ADR treatment reduced the expression of LINC00987 in HL-60 and MOLM13 cell (Supplementary Fig. 2). Thus, LINC00987 was chosen to study its effect on drug resistance in AML cells.

### LINC00987 downregulation reduces the ADR resistance of ADR-resistant AML cells

**Fig. 2:**
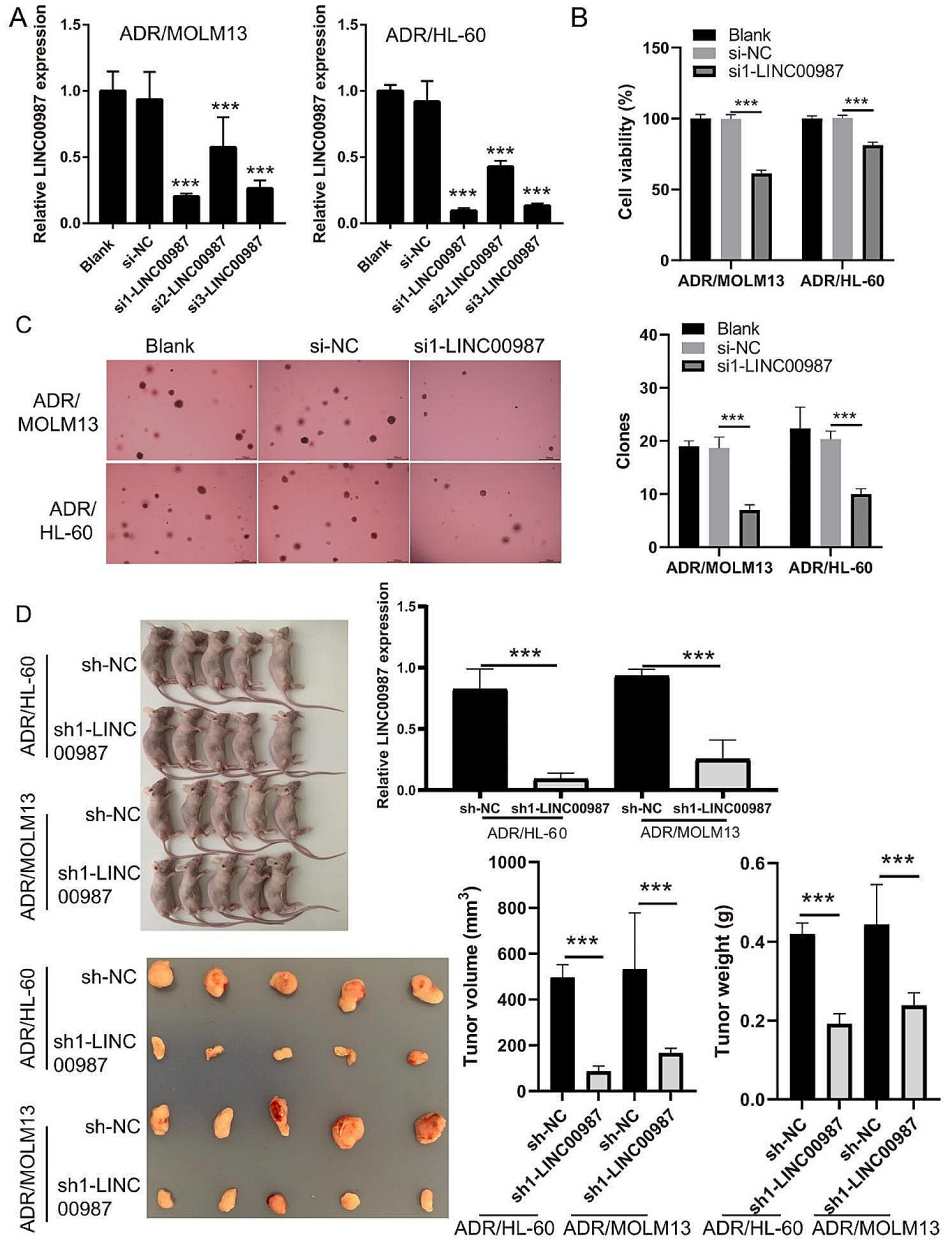
Decreased expression of LINC00987 reduces the cell viability and ADR-resistance of AML cells. (**A**) LINC00987 expression in ADR/MOLM13 and ADR/HL-60 cells was measured via RT-qPCR after siRNA transfection at 24 h. (**B**) The proliferation of ADR/MOLM13 and ADR/HL-60 cells was detected by CCK8 after siRNA transfection at 24 h under 0.2 µM ADR treatment. The cell viability was calculated according to proliferation. (**C**) Clone formation of ADR/MOLM13 and ADR/HL-60 cells was detected by soft agar colony formation assay. The number of cell clone formations was counted by imaging at 100x magnification. (D) Xenograft tumor was used to analyze the effect of LINC00987-silenced on the size of tumor growth. And LINC00987 expression in Xenograft tumor was measured via RT-qPCR. *P < 0.005 and ***P < 0.001

**Fig. 3:**
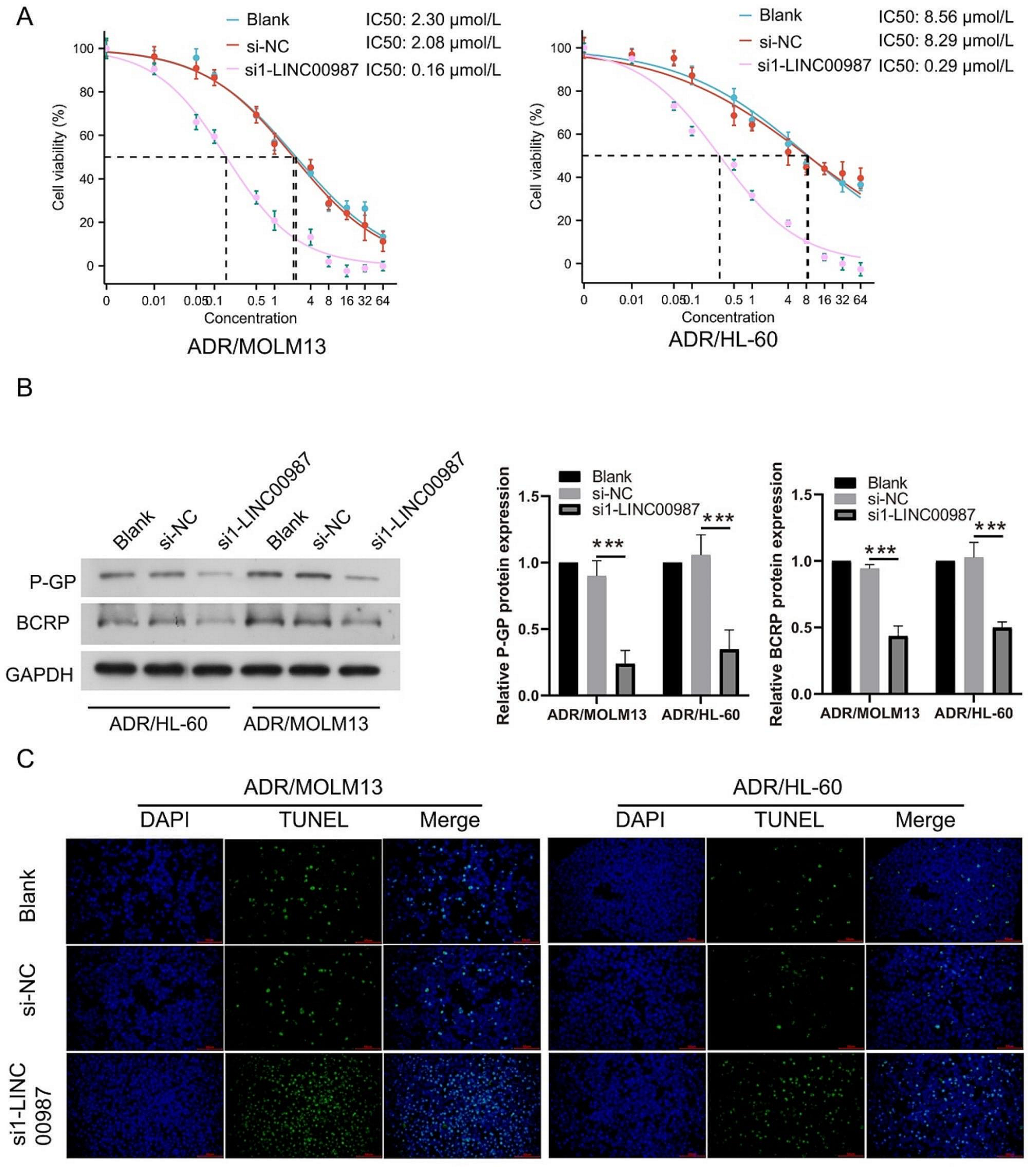
LINC00987 downexpression reduces the ADR resistance of ADR-resistant AML cells under 0.2 µM ADR treatment. (**A**) The IC50 of ADR for si1-LINC00987-transfected ADR/MOLM13 and ADR/HL-60 cells were shown after different concentrations of ADR treatment at 24 h. (**B**) Under 0.2 µM ADR treatment, P-GP and BCRP expression in ADR/MOLM13 and ADR/HL-60 cells were measured by western blotting. (**C**) Apoptosis of ADR/MOLM13 and ADR/HL-60 cells were detected by TUNEL staining and imaging at 400× magnification. ***P < 0.001

To detect the effect of LINC00987 on ADR-resistant AML cells, ADR/MOLM13 and ADR/HL-60 cells were transfected with LINC00987 siRNA and then treated with 0.2 µM (≈ IC 50 of MOLM13) ADR. LINC00987 expression in ADR/MOLM13 and ADR/HL-60 cells was significantly inhibited after transfection with siRNAs, especially si1-LINC00987 (Fig. 2A). Under 0.2 µM ADR treatment, the survival rate and the number of cell clone formation of ADR/MOLM13 and ADR/HL-60 cells in the si1-LINC00987 group was significantly lower than that in the si-NC group (Fig. 2B and C). In addition, compared to the sh-NC group, LINC00987 expression in xenograft tumor was were significantly decreased in sh1-LINC00987 group and the tumor volume were significantly decreased in sh1-LINC00987 group under 50 µM ADR treatment (Fig. 2D). The IC50 of si1-LINC00987-transfected ADR/MOLM13 (0.16 µmol/L) and ADR/HL-60 (0.29 µmol/L) cells were evidently reduced compared to the IC50 of si1-NC-transfected (2.08 µmol/L) and ADR/HL-60 (8.29 µmol/L) cells (Fig. 3A). The expression of the drug resistance proteins (P-GP and BCRP) in the si1-LINC00987 group was clearly lower than that in the si-NC group (Fig. 3B). More interestingly, under 0.2 µM ADR treatment, the apoptosis of ADR/MOLM13 and ADR/HL-60 cells in the si1-LINC00987 group was noticeably higher than that in si-NC group (Fig. 3C).

### LINC00987 overexpression enhances the ADR-resistance of AML cells

**Fig. 4:**
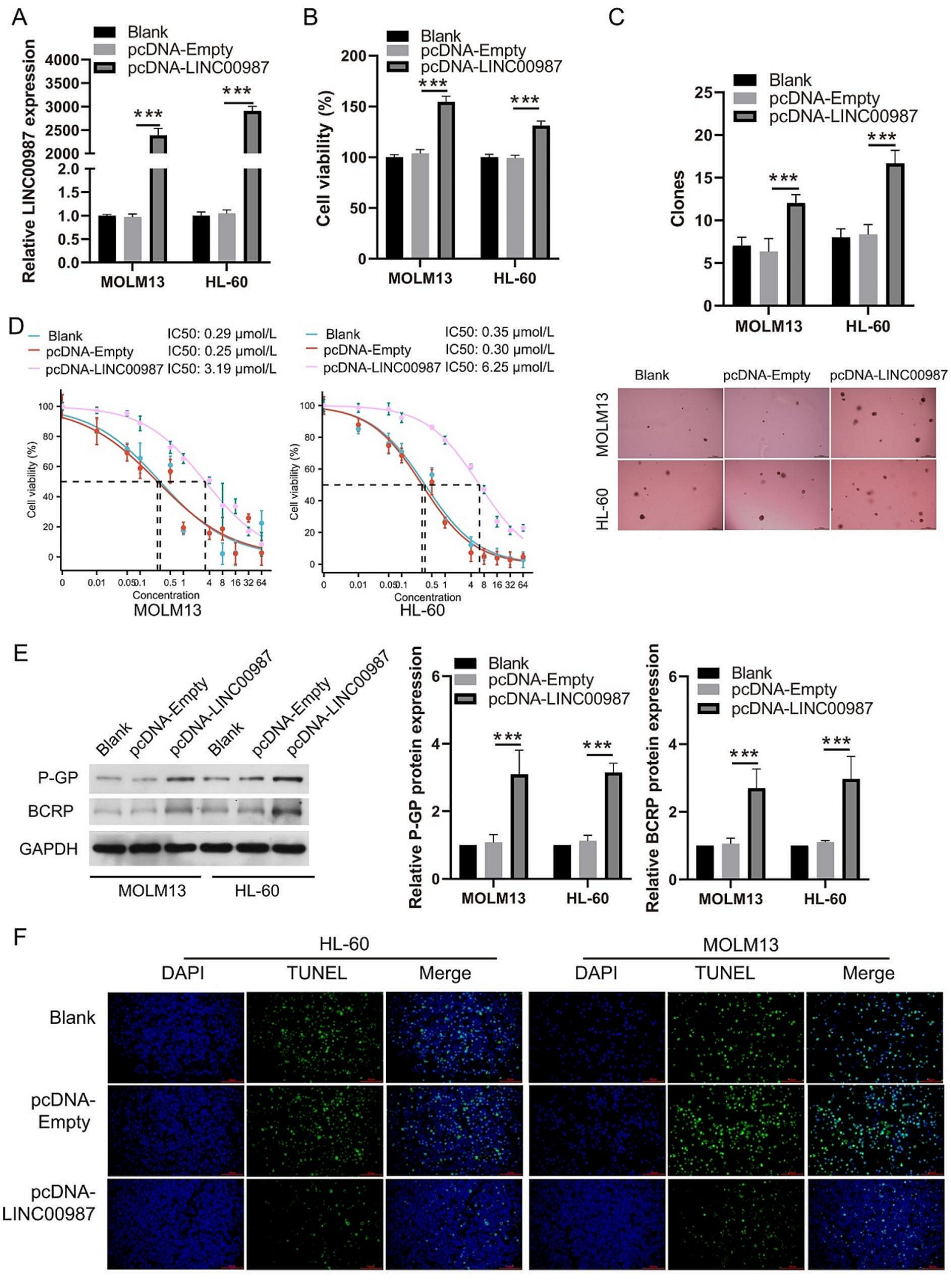
LINC00987 overexpression enhanced the cell viability, clone formation and ADR-resistance of AML cells. (**A**) LINC00987 expression in MOLM13 and HL-60 cells were measured by RT-qPCR after transfection with pcDNA-LINC00987 and pcDNA-Empty at 24 h. (**B**) The proliferation of MOLM13 and HL-60 cells was detected by CCK8 after transfection with pcDNA-LINC00987 and pcDNA-Empty at 24 h under 0.2 µM ADR treatment. Then, the cell viability was calculated according to proliferation. (**C**) Clone formation was detected by soft agar assay colony formation. The number of cell clone formations was counted by imaging at 100x magnification. (**D**) The IC50 of ADR for the pcDNA-LINC00987-transfected MOLM13 and HL-60 cells were shown after different concentrations of ADR treatment at 24 h. (**E**) Under 0.2 µM ADR treatment, P-GP and BCRP expression in MOLM13 and HL-60 cells were measured by Western blot. (**F**) Apoptosis was detected by TUNEL staining and imaging at 400x magnification. ***P < 0.001

To detect the effect of LINC00987 on ADR resistance in AML cells, MOLM13 and HL-60 cells which were transfected into pcDNA-LINC00987 and then treated with 0.2 µM ADR. LINC00987 expression in MOLM13 and HL-60 cells was significantly higher in the pcDNA-LINC00987 group than that in the pcDNA-empty group (Fig. 4A). Under 0.2 µmol/L ADR treatment, the cell viability and the number of cell clone formations of MOLM13 and HL-60 cells in pcDNA-LINC00987 group was significantly higher than that in pcDNA-Empty group (Fig. 4B and C). The IC50 of pcDNA-LINC00987-treanfected MOLM13 (3.19 µmol/L) and HL-60 (6.25 µmol/L) cells were evidently increased compared with the IC50 of MOLM13 (0.25 µmol/L, ) and HL-60 (0.30 µmol/L) cells (Fig. 4D). In addition, P-GP and BCRP expression in the pcDNA-LINC00987 group was visibly higher than that in the pcDNA-empty group (Fig. 4E). More interestingly, under 0.2 µM ADR treatment, the apoptosis of MOLM13 and HL-60 cells in the pcDNA-LINC00987 group was noticeably lower than that in pcDNA-Empty group (Fig. 4F).

### LINC00987 functions as a ceRNA of miR-4458 to regulate ADR resistance in ADR-resistant AML cells

To examine the molecular mechanism of LINC00987 in AML cells, bioinformatics websites, including miRcord and LncBase Predicted v2, were used to analyze the potential miRNA sponges of LINC00987. The results revealed six miRNAs (Supplementary Fig. 3A). qRT-PCR analysis demonstrated that miR-4458 expression in ADR/MOLM13 and ADR/HL-60 cells was abnormally reduced compared to that in MOLM13 and HL-60 cells, whereas the expression of the other five miRNAs was increased (Supplementary Fig. 3B). These results suggest that only miR-4458 has a potential negative association with ADR resistance in AML. The binding sites of LINC00987 and miR-4458 were analyzed by GEPIA and luciferase reporter assay demonstrated that luciferase activities were significantly reduced between the LINC00987-wt + miR-4458 mimic group and the LINC00987-wt + NC mimic group. However, no obvious inhibitory effect between the LINC00987-mut + miR-4458 mimic group and the LINC00987-mut + NC mimic group was observed (Supplementary Fig. 3C and 3D). Anti-Ago2 RIP assay showed that LINC00987 and miR-4458 expression in ADR/MOLM13 and ADR/HL-60 cells was significantly enriched in the anti-Ago2 group compared with the anti-IgG group (Supplementary Fig. 3E). Next, there is no significant correlation between the expression of LINC00987 and miR-4458 in AML patients (Supplementary Fig. 4A). These results indicate that LINC00987 functions as a ceRNA of miR-4458.

**Fig. 5:**
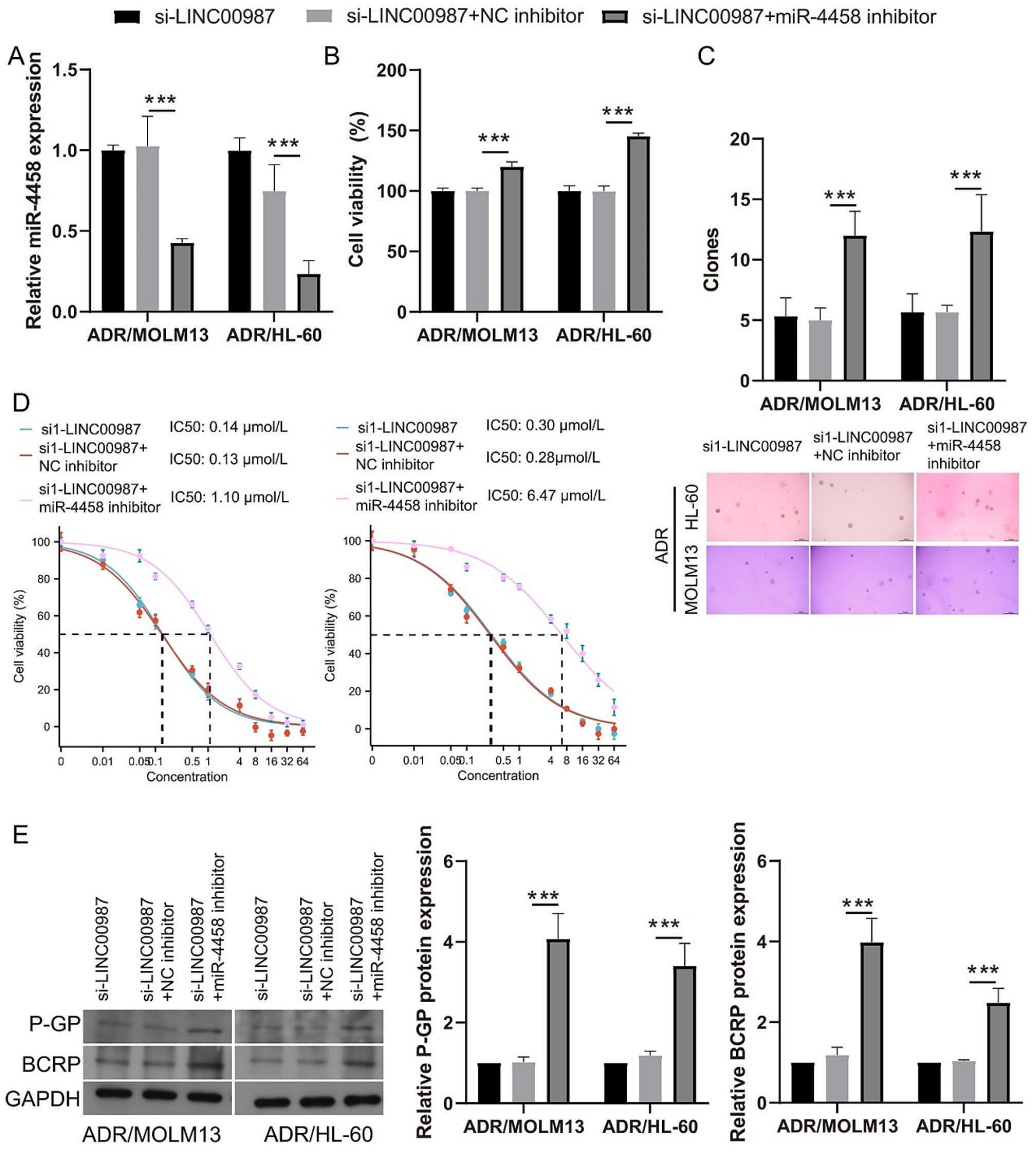
miR-4458 regulates cell viability and ADR-resistance to affect LINC00987 function in ADR-resistant AML cells. (**A**) miR-4458 expression in ADR/MOLM13 and ADR/HL-60 cells were measured via RT-qPCR after co-transfection at 24 h. (**B**) The proliferation of ADR/MOLM13 and ADR/HL-60 cells was detected by CCK8 after co-transfection at 24 h under 0.2 µM ADR treatment. Then, the cell viability was calculated according to proliferation. (**C**) Clone formation of ADR/MOLM13 and ADR/HL-60 cells was detected by soft agar colony formation assay after co-transfection at 24 h under 0.2 µM ADR treatment. The number of cell clone formations was counted by imaging at 100× magnification. (**D**) The IC50 of ADR for co-transfected ADR/MOLM13 and ADR/HL-60 cells were shown after different concentrations of ADR treatment at 24 h. (**E**) Under 0.2 µM ADR treatment, P-GP and BCRP expression in ADR/MOLM13 and ADR/HL-60 cells were measured by western blotting after si1-LINC00987 and miR-4458 inhibitor co-transfection. ***P < 0.001

To detect the effect of miR-4458 on ADR-resistant AML cells, ADR/MOLM13 and ADR/HL-60 cells were transfected with a miR-4458 mimic and then treated with 0.2 µM ADR. miR-4458 expression in ADR/MOLM13 and ADR/HL-60 cells was significantly enhanced after transfection with the miR-4458 mimic (Supplementary Fig. 5A). Under 0.2 µM ADR treatment, the cell viability and the number of cell clone formation of ADR/MOLM13 and ADR/HL-60 cells in the miR-4458 mimic group was significantly lower than that in the NC mimic group (Supplementary Fig. 5B and 55 C). The IC50 of miR-4458 mimic-transfected ADR/MOLM13 (0.27 µmol/L) and ADR/HL-60 (0.14 µmol/L) cells were visibly reduced compared with the IC50 of ADR/MOLM13 (2.40 µmol/L) and ADR/HL-60 (7.55 µmol/L) cells (Supplementary Fig. 5D). More interestingly, P-GP and BCRP expression in the miR-4458 mimic group was clearly lower than that in the NC mimic group (Fig. 5E).

Furthermore, to further clarify the relationship between LINC00987 and miR-4458 in ADR-resistant AML cells, si1-LINC00987 and the miR-4458 inhibitor were co-transfected into ADR/MOLM13 and ADR/HL-60 cells and then treated with 0.2 µM ADR. MiR-4458 expression in ADR/MOLM13 and ADR/HL-60 cells was significantly inhibited after co-transfection with si1-LINC00987 and the miR-4458 inhibitor (Fig. 5A). Under 0.2 µM ADR treatment, the cell viability and the number of cell clone formations of ADR/MOLM13 and ADR/HL-60 cells co-transfected with si1-LINC00987 and the miR-4458 inhibitor was significantly higher than that in the co-transfected si1-LINC00987 and NC inhibitor group (Fig. 5B and C). The IC50 of the si1-LINC00987 and miR-4458 inhibitor-co-transfected ADR/MOLM13 (1.1 µmol/L) and ADR/HL-60 (2.8 µmol/L) cells were noticeably increased compared with the IC50 of si1-LINC00987-transfected ADR/MOLM13 (0.16 µmol/L) and ADR/HL-60 (0.29 µmol/L) cells (Fig. 5D). More interestingly, P-GP and BCRP expression in the co-transfected with si1-LINC00987 and the miR-4458 inhibitor was clearly higher than that in the co-transfected si1-LINC00987 and NC inhibitor group (Fig. 5E).

### miR-4458 inhibits HMGA2 expression to affect LINC00987 function in ADR-resistant AML cells

To examine the target of miR-4458 in ADR-resistant AML cells, bioinformatics websites (including Starbase 3.0, miRBD, and Targetscan 8.0) were used to analyze the potential targets of miR-4458. In addition, the top 100 genes that had a similar expression trend to LINC00987 in AML were analyzed using GEPIA. The intersections between the four sets of results were calculated; HMGA2 was found at this intersection (Supplementary Fig. 6A). The binding sites of LINC00987 and miR-4458 are shown in Supplementary Fig. 6B. In addition, luciferase reporter assays demonstrated that the luciferase activities were significantly reduced between the HMGA2 3’UTR-wt + miR-4458 mimic group and the HMGA2 3’UTR-wt + NC mimic group. However, there was no obvious inhibitory change between the HMGA2 3’UTR-mut + miR-4458 mimic group and HMGA2 3’UTR-mut + NC mimic group (Supplementary Fig. 6C). These results showed that miR-4458 can bind to the HMGA2 3’-UTR in ADR/MOLM13 and ADR/HL-60 cells. In addition, HMGA2 protein expression in ADR/MOLM13 and ADR/HL-60 cells was notably reduced in the si-LINC00987 group compared to the si-NC group (Supplementary Fig. 6D), while HMGA2 protein expression in MOLM13 and HL-60 cells was notably enhanced in the pcDNA-LINC00987 group compared to the pcDNA-Empty group (Supplementary Fig. 6D). Moreover, HMGA2 protein expression in ADR/MOLM13 and ADR/HL-60 cells was notably enhanced in the si-LINC00987 + miR-4458 inhibitor group compared to the si-LINC00987 + NC inhibitor group, while it was notably reduced in the miR-4458 mimic group compared to the NC mimic group (**Supplementary Fig. 6E**). Next, there is no significant correlation between the expression of HMGA2 mRNA and miR-4458 in AML patients (Supplementary Fig. 4B). These results showed that HMGA2 protein expression was inhibited by miR-4458 and promoted by LINC00987.

**Fig. 6:**
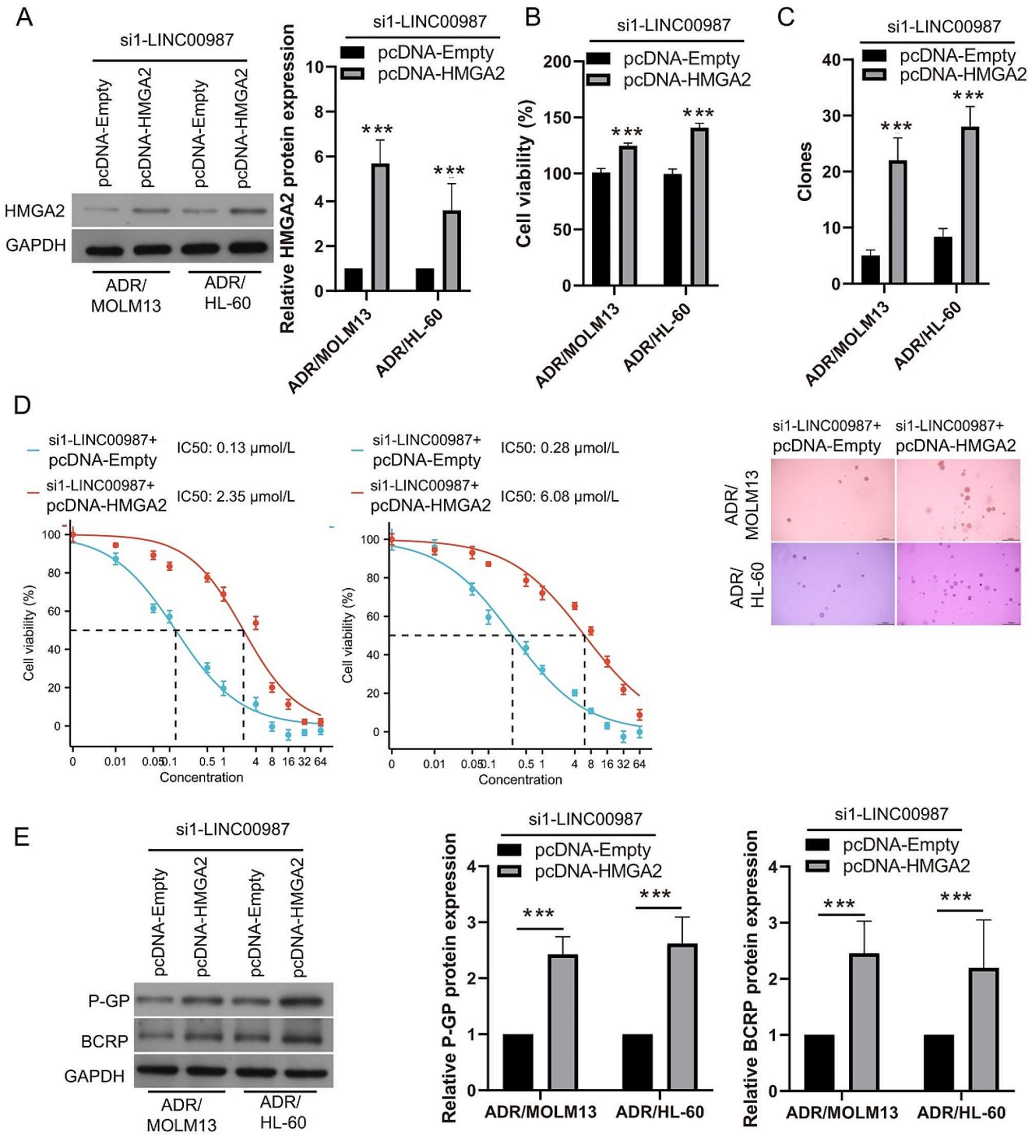
HMGA2 promotes cell viability and ADR-resistance to assist in LINC00987 function in ADR-resistant AML cells. (**A**) HMGA2 protein expression in ADR/MOLM13 and ADR/HL-60 cells were measured by Western blot after si1-LINC00987 and pcDNA-HMGA2 co-transfection at 24 h. (**B**) The proliferation of ADR/MOLM13 and ADR/HL-60 cells was detected using CCK8 after co-transfection at 24 h under 0.2 µM ADR treatment. Then, the cell viability was calculated according to proliferation. (**C**) Clone formation of ADR/MOLM13 and ADR/HL-60 cells was detected by soft agar colony formation assay after co-transfection at 24 h under 0.2 µM ADR treatment. The number of cell clone formations was counted by imaging at 100× magnification. (**D**) The IC50 of ADR for co-transfected ADR/MOLM13 and ADR/HL-60 cells were shown after different concentrations of ADR treatment at 24 h. (**E**) Under 0.2 µM ADR treatment, P-GP and BCRP expression in ADR/MOLM13 and ADR/HL-60 cells were measured by western blotting after si1-LINC00987 and pcDNA-HMGA2 co-transfection. ***P < 0.001

To further clarify the relationship between LINC00987 and HMGA2 in ADR-resistant AML cells, si1-LINC00987 and pcDNA-HMGA2 were co-transfected into ADR/MOLM13 and ADR/HL-60 cells and then treated with 0.2 µM ADR. HMGA2 expression in ADR/MOLM13 and ADR/HL-60 cells was significantly enhanced after co-transfection with si1-LINC00987 and pcDNA-HMGA2 (Fig. 6A). Under 0.2 µM ADR treatment, the cell viability and the number of cell clone formations of ADR/MOLM13 and ADR/HL-60 cells in the co-transfected si1-LINC00987 and pcDNA-HMGA2 group was significantly higher than that in the co-transfected si1-LINC00987 and pcDNA-Empty group (Fig. 6B and C). The IC50 of the si1-LINC00987 and pcDNA-HMGA2-co-transfected ADR/MOLM13 (2.35 µmol/L) and ADR/HL-60 (6.08 µmol/L) cells were evidently increased compared with the IC50 of the si1-LINC00987-transfected ADR/MOLM13 (0.13 µmol/L) and ADR/HL-60 (0.28 µmol/L) cells (Fig. 6D). More interestingly, P-GP and BCRP expression in the co-transfected with si1-LINC00987 and pcDNA-HMGA2 was clearly higher than that in the co-transfected si1-LINC00987 and pcDNA-Empty group (Fig. 6E).

## Discussion

Only small number of aberrant lincRNA, such as LINC00239, has been found to regulate AML ADR-resistance according to experimentally and functionally validated [19]. Chemotherapy resistance is a major barrier in treating AML patients and improving outcomes in patients with AML [20, 21]. Therefore, identifying targets to improve the chemoresistance of AML is of great significance for AML treatment. Herein, high LINC00987 expression was found in AML patients and indicates poor prognosis. In addition, our data demonstrated LINC00987 expression in ADR-resistance AML cell was higher than that in ADR-sensitive AML cell. Furthermore, LINC00987 downregulation reduced the proliferation, clone formation, IC 50, drug resistance proteins (P-GP and BCRP), and the volume of Xenograft tumor where promoted apoptosis under ADR treatment in ADR-resistance AML cell, while LINC00987 overexpression in ADR-sensitive AML cell played the opposite role. This study suggested that LINC00987 overexpression enhanced ADR-resistance. Furthermore, LINC00987 enhanced HMGA2 protein by sponging miR-4458 to contribute to ADR-resistance (Supplementary Fig. 7).

Recently, emerging studies had illustrated the oncogenic role of LINC00987 in breast cancer and osteosarcoma [22, 23]. Importantly, high LINC00987 expression is associated with poor prognosis in AML patients [24]. The previous study was supported by our study indicating that LINC00987 expression was increased in AML patients and associated with poor prognosis using public databases analysis. Furthermore, this study firstly demonstrated that LINC00987 expression in ADR-resistance AML cell was prominently higher than that in ADR-sensitive AML cell, and LINC00987 downregulation reversed ADR-resistance in AML cells. Additionally, LINC00987 knockdown have been reported to inhibit the progression of AML cells via inhibiting proliferation and promoting apoptosis, showed that LINC00987 is an oncogene that promotes AML development [25]. Combined with our study, LINC00987 predicts poor prognosis, which is closely related not only to its ability to regulate the progression of AML, but also to its ability to regulate ADR-resistance in AML. This is the only study to clarify the function of LINC00987 in ADR-resistance, contributing to thestudy and promotion of LINC00987 in other ADR resistant cancers.

LincRNAs often act as endogenous ceRNAs to bind miRNAs by competing with miRNA-targeted genes [26]. For example, LINC00987 binds to miR-376a-5p by competing with FNBP1 to promote osteosarcoma cell development [23]. This study identified that LINC00987 acts as an endogenous sponge of miR-4458 in ADR-resistance AML cells. Emerging studies have illustrated that miR-4458 serves as the tumor suppressor miRNA in NSCLC, melanoma, and liver cancer by regulating tumorigenesis, epithelial-mesenchymal transition, and the immune microenvironment [27–29]. In addition, miR-4458 attenuates chemoresistance in NSCLC, breast cancer, and gastric cancer [30–32]. Similarly, our study showed that miR-4458 expression was reduced in ADR-resistance AML cell than that in ADR-sensitive AML cell and miR-4458 overexpression attenuated ADR resistance. Interestingly, miR-4458 expression is low in AML tissues, and miR-4458 downregulation promotes the proliferation and invasion of AML cells [33]. Combined with our study, miR-4458 inhibits the growth, development, and drug resistance of AML, making it an ideal target for the treatment of AML. In addition, this study also found that miR-4458 downregulation reversed the effect of LINC00987 knockdown on ADR resistance, suggesting that LINC00987 functions as a ceRNA of miR-4458 to regulate ADR resistance in ADR-resistant AML cells.

Finally, this study found that miR-4458 could bind to the 3’-UTR of HMGA2. Previous study found that HMGA2 is predominantly highly expressed in AML tissues and independently predicts poor prognosis in AML [34]. HMGA2 promotes cell proliferation, AML progression, and reduced chemosensitivity [35, 36]. The results of those previous study were supported to the result of our study, which also indicate that HMGA2 overexpression enhanced ADR resistance in AML cells. Notably, HMGA2 expression was regulated by miRNA and lncRNA in many cancers [37, 38]. Here, LINC00987 knockdown and miR-4458 overexpression reduced HMGA2 expression in ADR-resistance AML cell. Most importantly, HMGA2 overexpression reversed the effect of LINC09987 silencing in suppressing ADR resistance in ADR-resistant AML cells. This is the first study to reveal that HMGA2 was regulated by LINC09987/miR-4458 axis to enhance ADR resistance in AML cell.

## Conclusion

Functionally, downregulation of LINC00987 weakens ADR resistance by releasing miR-4458 to deplete HMGA2 in AML cells. This study contributes to a better understanding of the function of LINC00987 in chemoresistance of AML cells. Therefore, this study showed new light on the regulation of ADR resistance and indicated that LINC00987 may serve as a therapeutic target for the treatment of chemoresistant AML cells.

### Electronic supplementary material

Below is the link to the electronic supplementary material.

Supplementary Material 1

Supplementary Material 2

Supplementary Material 3

## Data Availability

The data used and/or analyzed during the current study are included in this published article.

## Abbreviations

ADR: Adriamycin
AML: Acute myeloid leukemia
BCRP: breast cancer resistance protein
ceRNAs: competitive endogenous RNAs
HMGA2: high mobility group AT-hook 2
P-GP: P glycoprotein
