# Supplementary material for: Silencing LINC00987 ameliorates adriamycin resistance of acute myeloid leukemia via miR-4458/HMGA2 axis
Source: Biol Direct. 2024 Jun 24;19:49. doi: 10.1186/s13062-024-00490-1 (PMC11195003; doi:10.1186/s13062-024-00490-1)
Supplement: Supplementary file 1 — Supplementary Material 1 [file 13062_2024_490_MOESM1_ESM.pdf]

Table 1 The primers for qPCR.

Target	Primers (5′-3′)
LINC00152-F	CCGGAATGCAGCTGAAAGAT
LINC00152-R	CTGGCCAGACAAATGGGAAA
LINC00982-F	AGTCGGGTGGAGATGTCTAG
LINC00982-R	AGACACCCTCCTGTAGATCTG
LINC00987-F	GTTCTGATTTCCTGGGTTACC
LINC00987-R	TTCCTAGAACTCACCCTTCCT
β-actin-F	GCATGGGTCAGAAGGATTCCT
β-actin-R	TCGTCCCAGTTGGTGACGAT
miR-98-5p-F	ACACTCCAGCTGGGTGAGGTAGTAAGTTGTA
miR-98-5p-R	CTCAACTGGTGTCGTGGA
miR-4458-F	ACACTCCAGCTGGGAGAGGTAGGTGTGG
miR-4458-R	CTCAACTGGTGTCGTGGA
miR-4500-F	ACACTCCAGCTGGGTGAGGTAGTAGTT
miR-4500-R	CTCAACTGGTGTCGTGGA
miR-125a-5p-F	ACACTCCAGCTGGGTCCCTGAGACCCTTTAACC
miR-125a-5p-R	CTCAACTGGTGTCGTGGA
miR-125b-5p-F	ACACTCCAGCTGGGTCCCTGAGACCCTAACT
miR-125b-5p-R	CTCAACTGGTGTCGTGGA
miR-4319-F	ACACTCCAGCTGGGTCCCTGAGCA
miR-4319-R	CTCAACTGGTGTCGTGGA
U6-F	CTCGCTTCGGCAGCACA
U6-R	AACGCTTCACGAATTTGCGT

F: forward primer; R: reverse primer.
