# Supplementary material for: Silencing LINC00987 ameliorates adriamycin resistance of acute myeloid leukemia via miR-4458/HMGA2 axis
Source: Biol Direct. 2024 Jun 24;19:49. doi: 10.1186/s13062-024-00490-1 (PMC11195003; doi:10.1186/s13062-024-00490-1)

Independent repetition 1

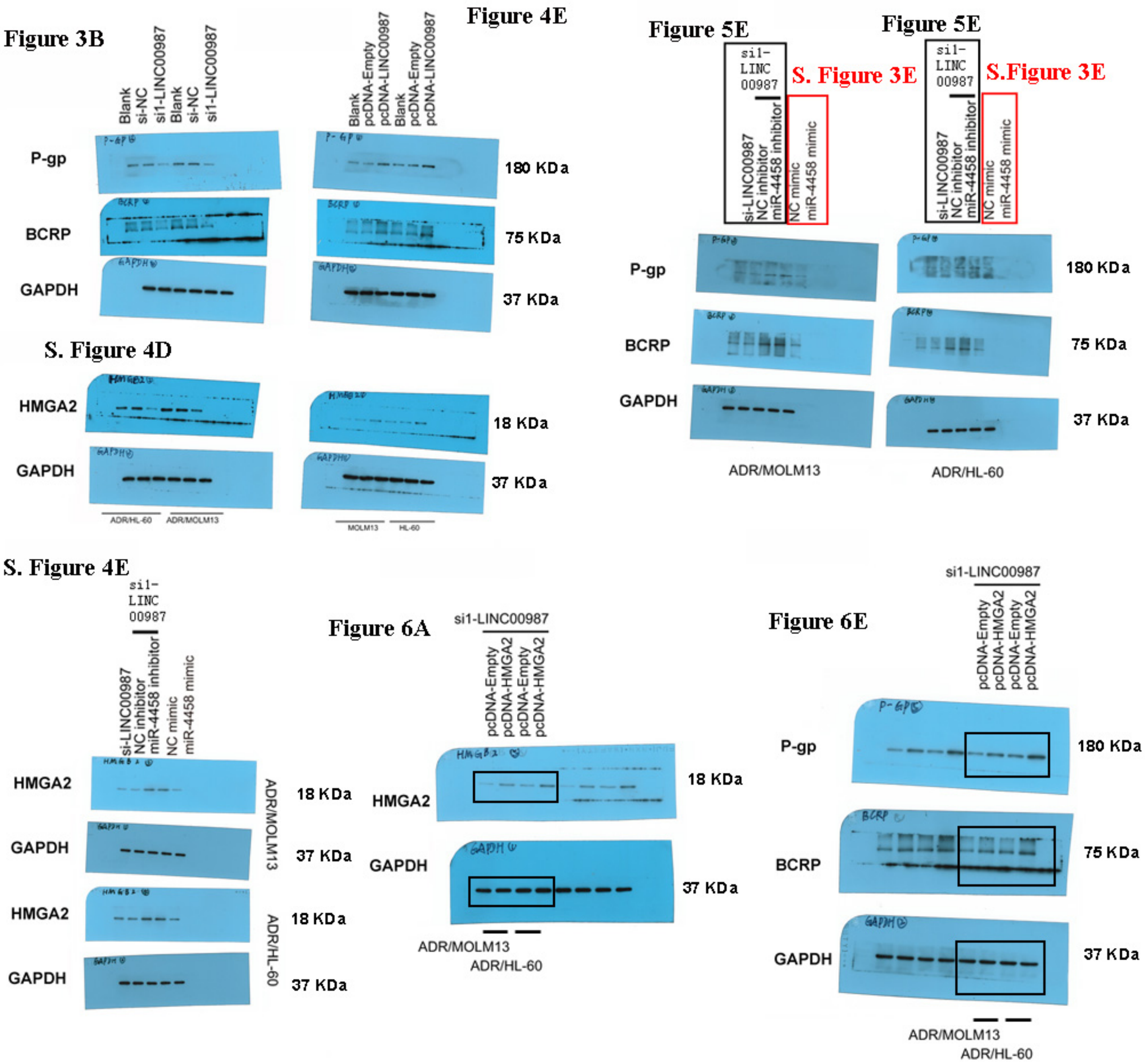

Independent repetition 2

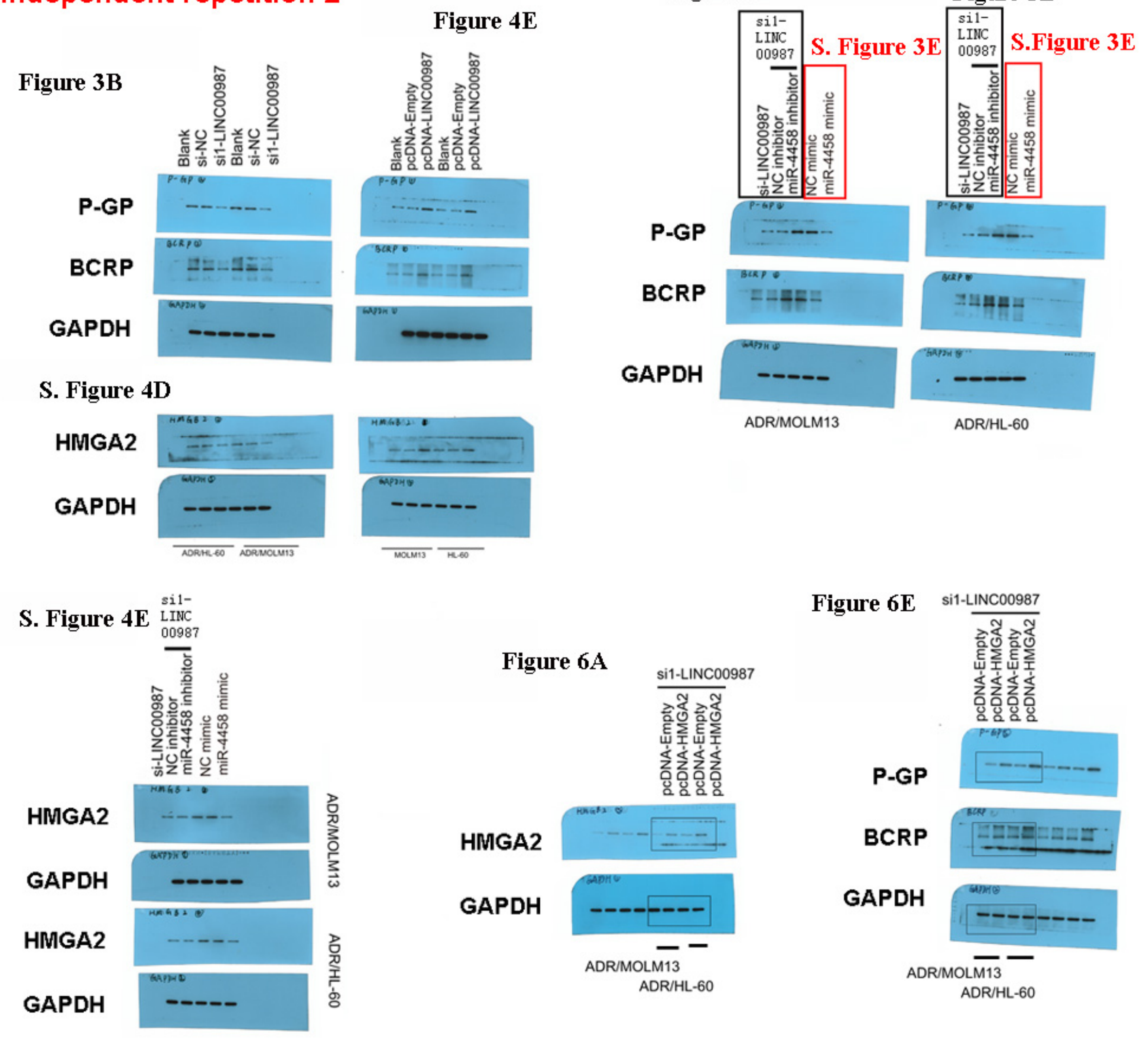

Independent repetition 3

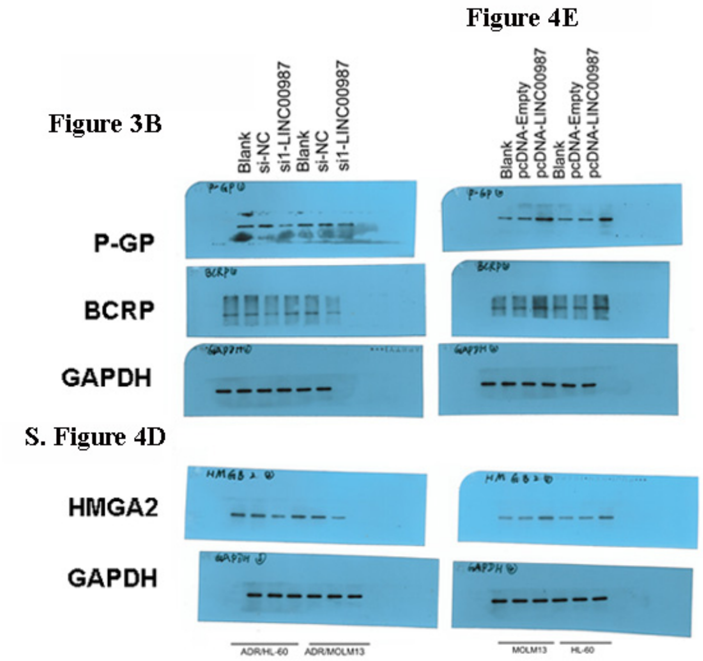

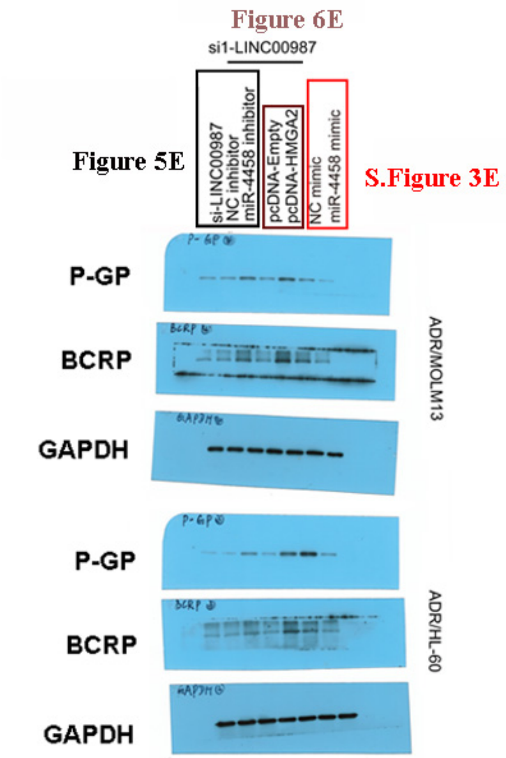

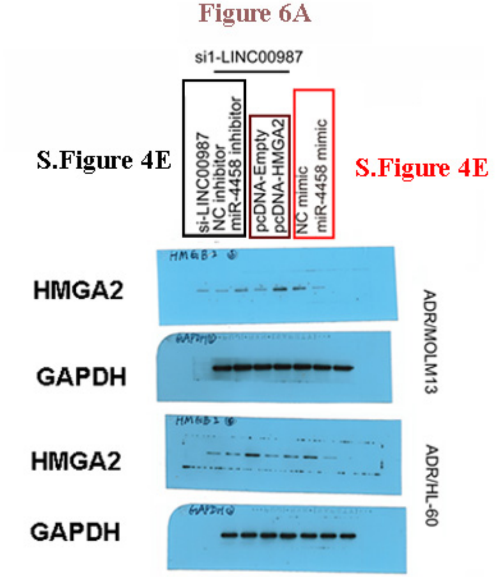

Supplement: Supplementary file 2 — Supplementary Material 2 [file 13062_2024_490_MOESM2_ESM.pdf]
