# Supplementary material for: Silencing LINC00987 ameliorates adriamycin resistance of acute myeloid leukemia via miR-4458/HMGA2 axis
Source: Biol Direct. 2024 Jun 24;19:49. doi: 10.1186/s13062-024-00490-1 (PMC11195003; doi:10.1186/s13062-024-00490-1)
Supplement: Supplementary file 3 — Supplementary Material 3 [file 13062_2024_490_MOESM3_ESM.pdf]

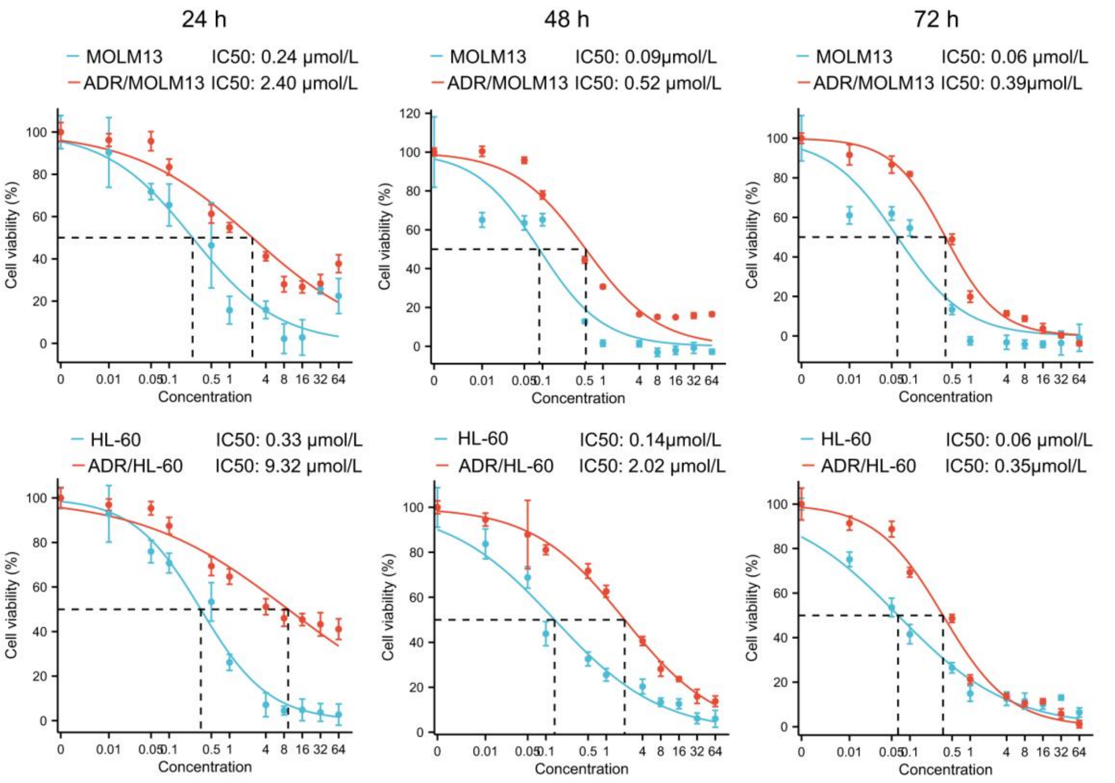

Supplementary Fig. 1 IC50 in ADR-resistant AML cells were increased compared with the corresponding AML cells. The cell viability of ADR for ADR-resistant AML cells (ADR/MOLM13 and ADR/HL-60 cell) and the corresponding AML cells (MOLM13 and HL-60) were calculated according to CCK8 results and IC50 were calculated after treatment with different concentrations of ADR.

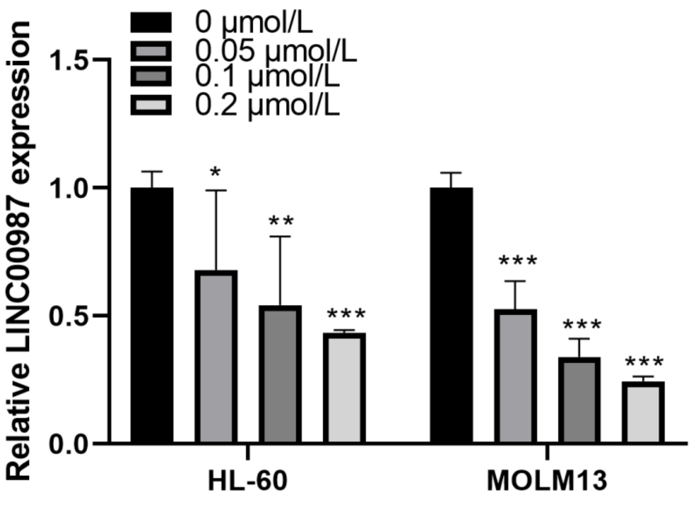

Supplementary Fig. 2 ADR treatment reduced the expression of LINC00987 in AML cell. After 24 hours of treatment with different concentrations of ADR, qRT PCR was used to detect the expression of LINC00987 in AML cells (HL-60 and MOLM13 cell). *P<0.05, **P<0.01; ***P<0.001.

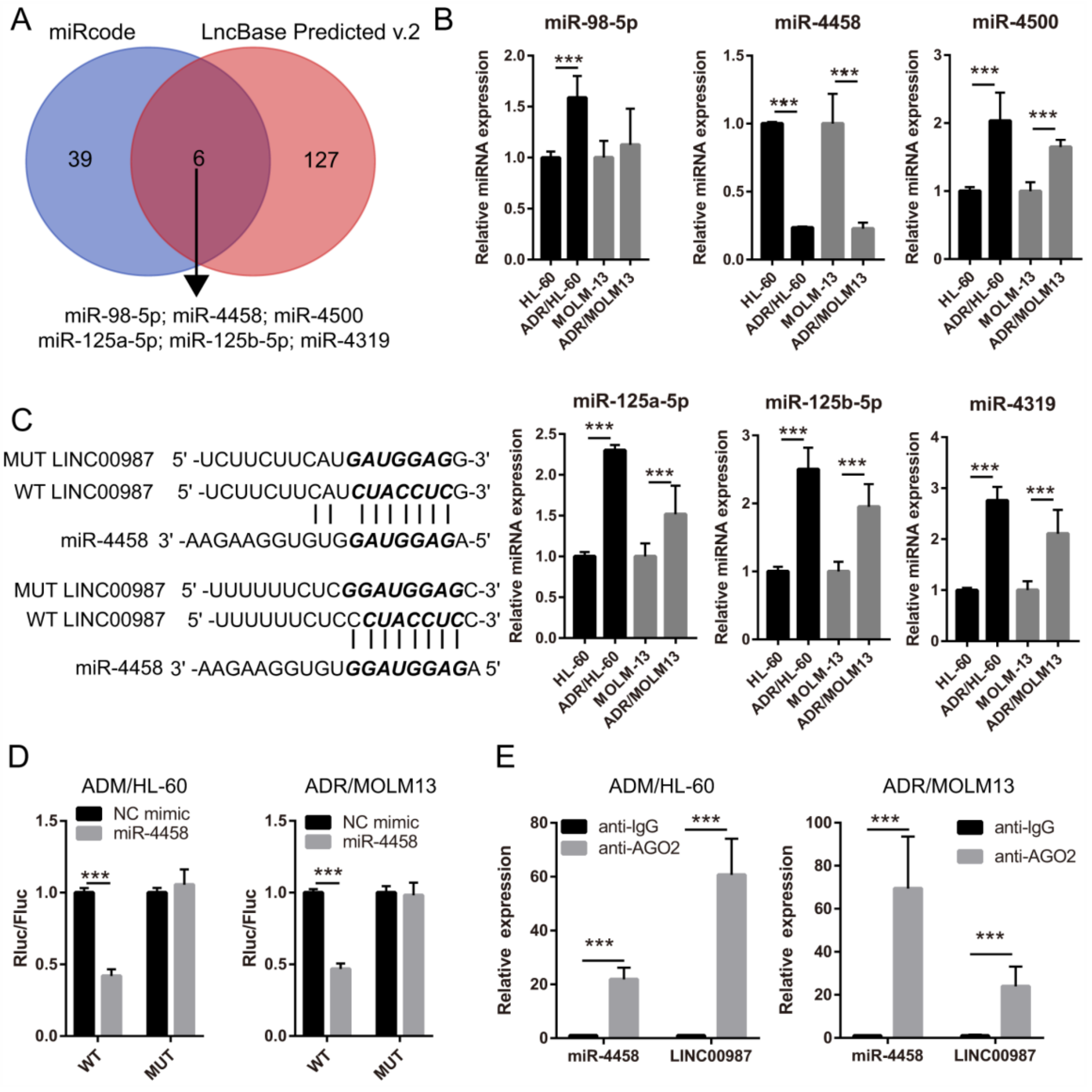

Supplementary Fig. 3 Direct binding of LINC00987 to miR-4458. (A) miRcord and LncBase Predicted v2 were used to analyze the potential miRNA sponges of LINC00987. (B) The expression of potential miRNA sponges in the ADR/MOLM13 and ADR/HL-60 cells were measured by qRT-PCR. (C) The binding sites between LINC00987 and miR-4458. (D) Luciferase activities were measured using a luciferase reporter assay. (E) LINC00987 and miR-4458 expression was enriched by Ago2 RIP assay and measured using RT-qPCR. ***P<0.001.

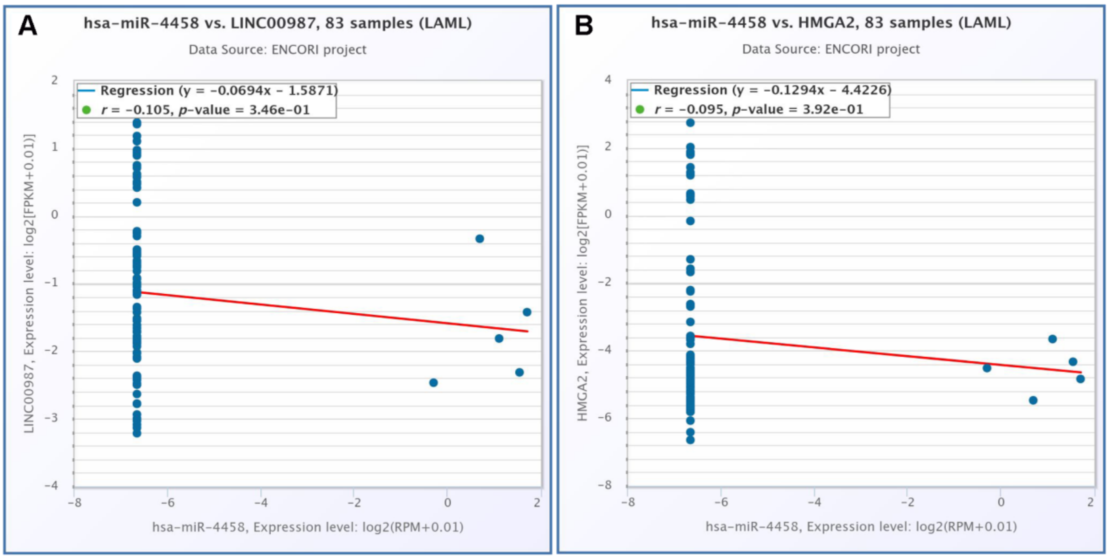

Supplementary Fig. 4 The clinical correlation of LINC00987 vs miR-4458 or HMGA2 v miR-4458 in AML patients were analyzed by Starbase 3.0. (A) The clinical correlation of LINC00987 vs miR-4458 were analyzed by Starbase 3.0; (B) The clinical correlation of HMGA2 v miR-4458 in AML patients were analyzed by Starbase 3.0

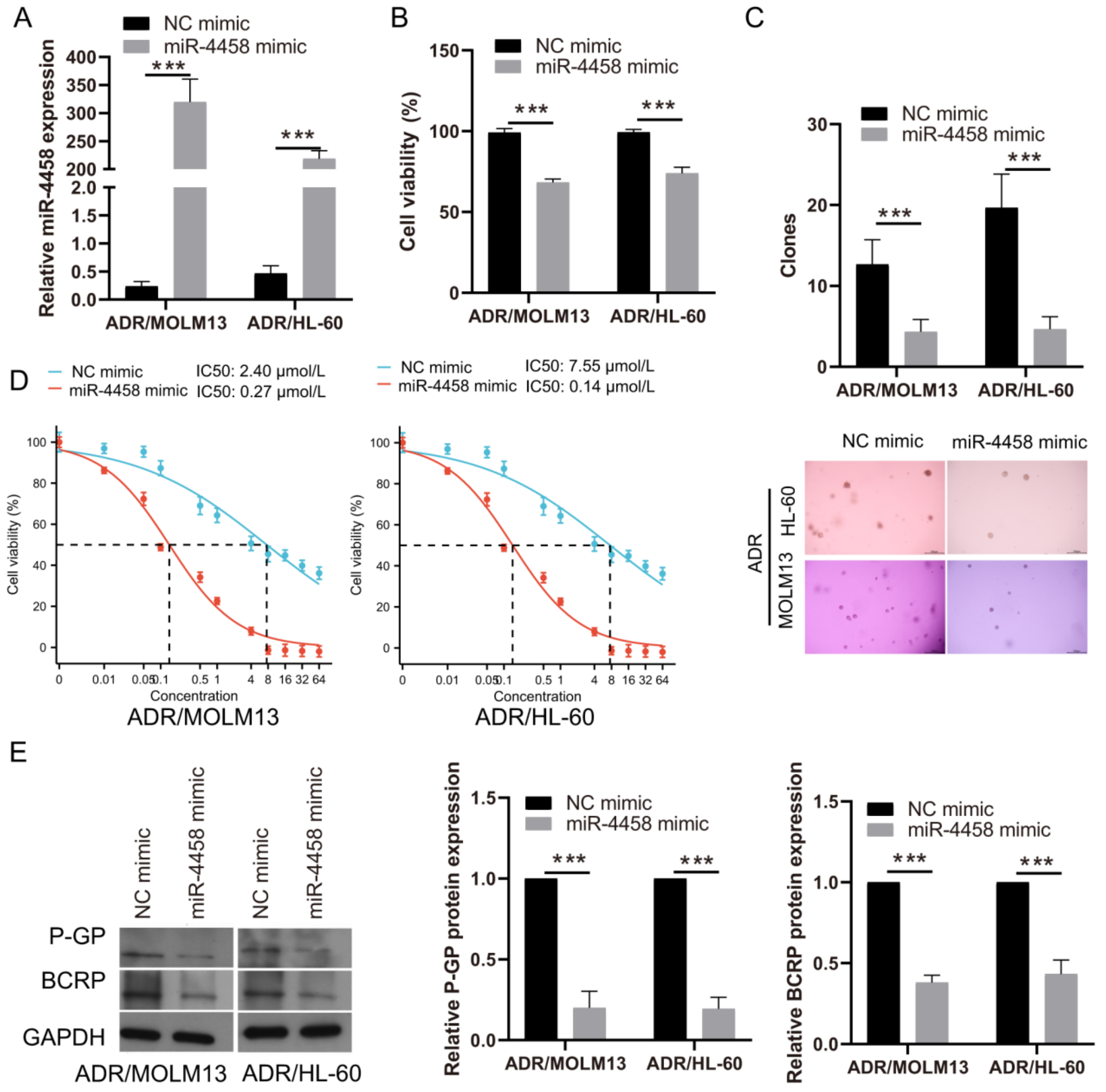

Supplementary Fig. 5 miR-4458 overexpression reduces the ADR resistance of ADR-resistant AML cells under 0.2 μM ADR treatment. (A) miR-4458 expression in ADR/MOLM13 and ADR/HL-60 cells was measured via RT-qPCR after miR-4458 mimic transfection at 24 h. (B) The proliferation of ADR/MOLM13 and ADR/HL-60 cells was detected by CCK8 after miR-4458 mimic transfection at 24 h under 0.2 μM ADR treatment. The cell viability was calculated according to proliferation. (C) Clone formation of ADR/MOLM13 and ADR/HL-60 cells was detected by soft agar colony formation assay. The number of cell clone formations was counted by imaging at 100x magnification. (E) Under 0.2 μM ADR treatment, P-GP and BCRP expression in ADR/MOLM13 and ADR/HL-60 cells were measured by western blotting. ***P<0.001

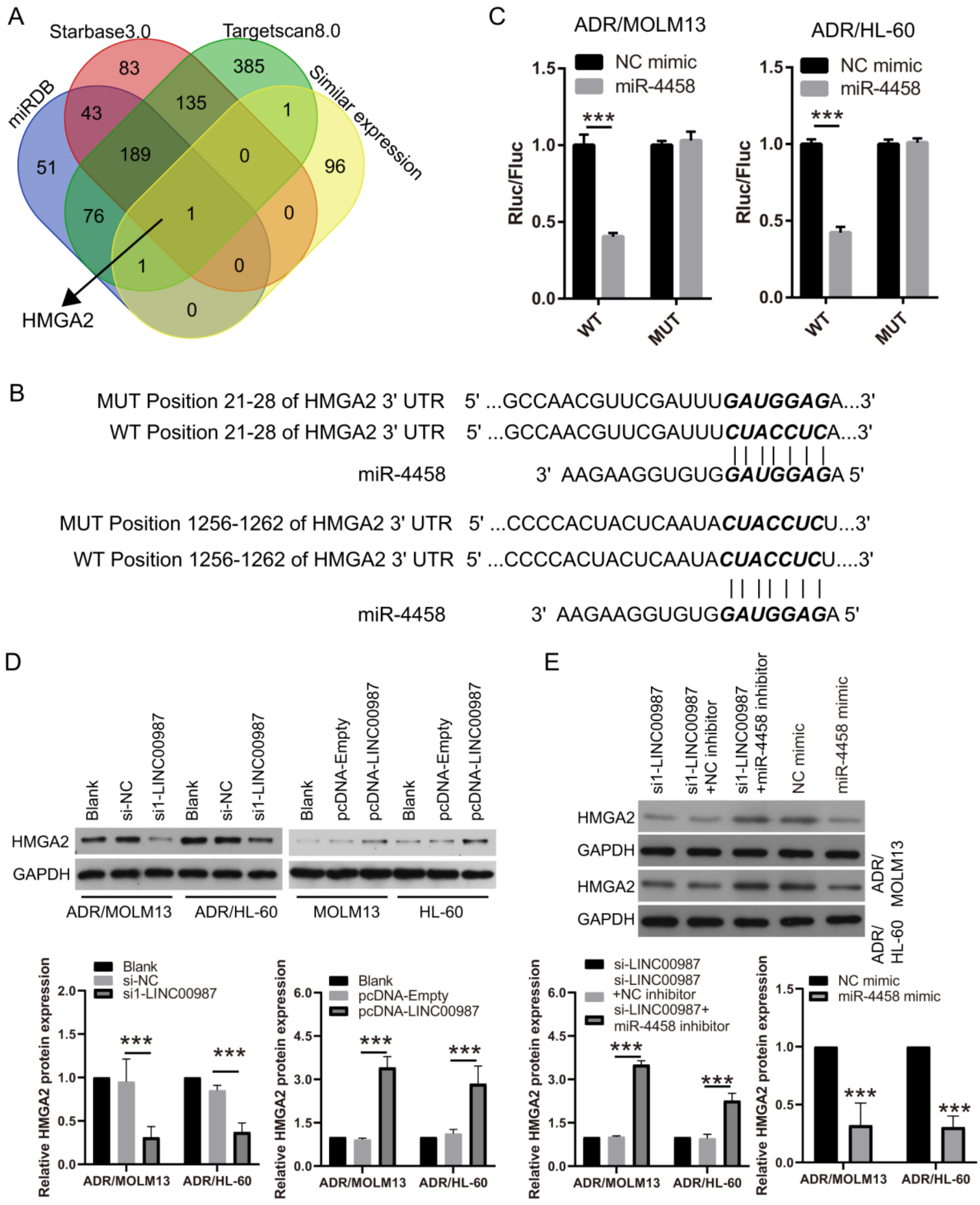

Supplementary Fig. 6 miR-4458 can bind with the 3'UTR of HMGA2 to inhibit HMGA2 expression in ADR-resistant AML cells. (A) Bioinformatics websites (including strabase 3.0, miRBD, and Targetscan 8.0) were used to analyze the potential targets of miR-4458. In addition, the top 100 genes which had a similar expression trend to LINC00987 in AML were analyzed by GEPIA. The intersections were calculated between the four results. (B) The binding sites between the 3'UTR of HMGA2 and miR-4458. (C) Luciferase activities were measured using a luciferase reporter assay. (D-E) HMGA2 protein expression was analyzed via Western blot in MOLM13,

HL-60, ADR/MOLM13, and ADR/HL-60 cells after transfection. ***P<0.001.

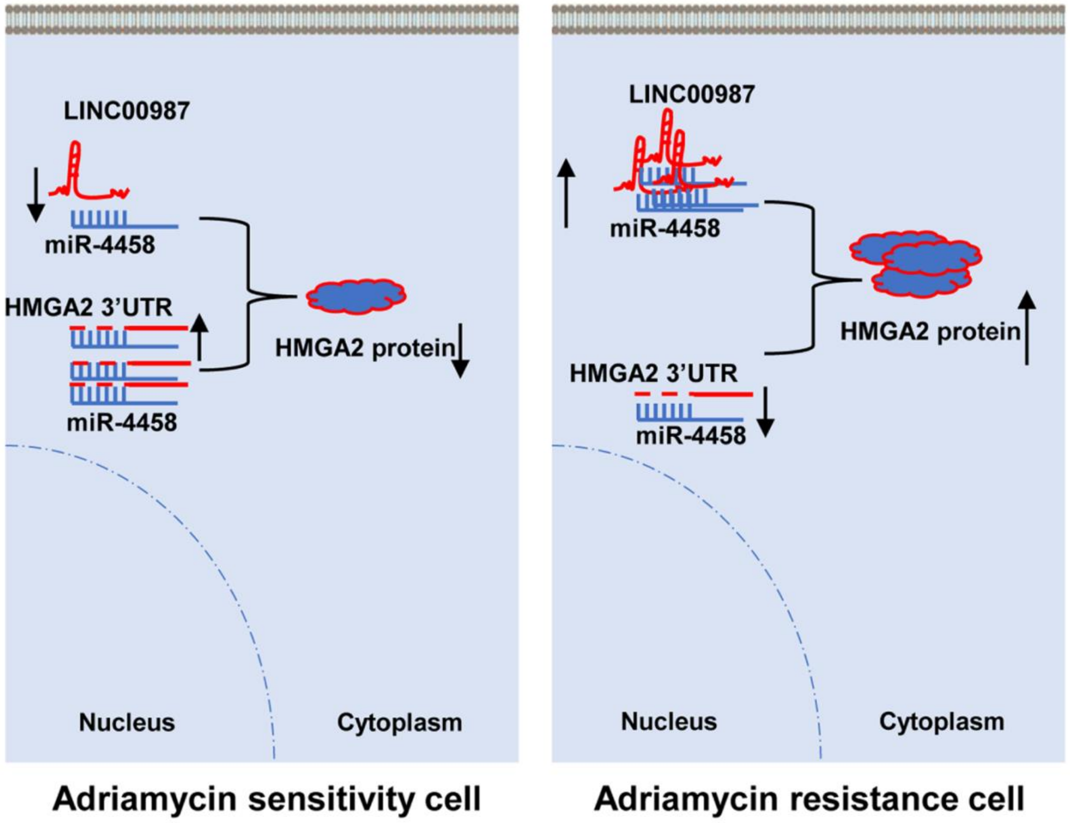

Supplementary Fig. 7 Graphic illustration of LINC00987 modulating the ADR resistance of AML cell. In doxorubicin sensitive and drug-resistant AML cells, LINC00987 and HMGA2 3 '-UTR competitively bind to miR-4458 through ceRNA mechanism. In doxorubicin resistant cells, the expression of LINC00987 increased, resulting in the increased binding of LINC00987 and miR-4458, while the binding of HMGA2 3 '- UTR and miR-4458 decreased. Finally, the level of HMGA2 protein increased, resulting in doxorubicin resistance; In doxorubicin sensitive cells, the expression of LINC00987 decreased, resulting in the decreased binding of LINC00987 and miR-4458 and the increased binding of HMGA2 3 '- UTR and miR-4458, which finally led to the decreased level of HMGA2 protein. Therefore, cells are sensitive to adriamycin treatment.
